# Chilli Anthracnose: The Epidemiology and Management

**DOI:** 10.3389/fmicb.2016.01527

**Published:** 2016-09-30

**Authors:** Amrita Saxena, Richa Raghuwanshi, Vijai Kumar Gupta, Harikesh B. Singh

**Affiliations:** ^1^Department of Botany, Banaras Hindu UniversityVaranasi, India; ^2^Department of Botany, Mahila Mahavidyalaya, Banaras Hindu UniversityVaranasi, India; ^3^Molecular Glycobiotechnology Group, Discipline of Biochemistry, National University of IrelandGalway, Ireland; ^4^Department of Mycology and Plant Pathology, Institute of Agricultural Sciences, Banaras Hindu UniversityVaranasi, India

**Keywords:** anthracnose, *Capsicum* spp., *Colletotrichum capsici*, epidemiology, disease management, biocontrol

## Abstract

Indian cuisine is renowned and celebrated throughout the world for its spicy treat to the tongue. The flavor and aroma of the food generated due to the use of spices creates an indelible experience. Among the commonly utilized spices to stimulate the taste buds in Indian food, whole or powdered chilli constitutes an inevitable position. Besides being a vital ingredient of of Indian food, chilli occupy an important position as an economic commodity, a major share in Indian economy. Chilli also has uncountable benefits to human health. Fresh green chilli fruits contain more Vitamin C than found in citrus fruits, while red chilli fruits have more Vitamin A content than as found in carrots. The active component of the spice, Capsaicin possesses the antioxidant, anti-mutagenic, anti-carcinogenic and immunosuppressive activities having ability to inhibit bacterial growth and platelet aggregation. Though introduced by the Portuguese in the Seventeenth century, India has been one of the major producers and exporters of this crop. During 2010–2011, India was the leading exporter and producer of chilli in the world, but recently due to a decline in chilli production, it stands at third position in terms of its production. The decline in chilli production has been attributed to the diseases linked with crop like anthracnose or fruit rot causing the major share of crop loss. The disease causes severe damage to both mature fruits in the field as well as during their storage under favorable conditions, which amplifies the loss in yield and overall production of the crop. This review gives an account of the loss in production and yield procured in chili cultivation due to anthracnose disease in Indian sub-continent, with emphasis given to the sustainable management strategies against the conventionally recommended control for the disease. Also, the review highlights the various pathogenic species of *Colletotrichum* spp, the causal agent of the disease, associated with the host crop in the country. The information in the review will prove of immense importance for the groups targeting the problem, for giving a collective information on various aspects of the epidemiology and management of the disease.

## Introduction

Chilli (*Capsicum annum* L.) is one of the most important constituent of the cuisines of tropical and subtropical countries and the fourth major crop cultivated globally. Around 400 different varieties of chilies are cultivated throughout the globe. The hottest variety being “Carolina Reaper” developed by a grower Ed Currie of West Indies having the maximum pungency of about 2.2 million SHU (Scoville Heat Units; PuckerButt Pepper Company, 2013). One of the hot chilli varieties of the world “Naga Jalokia,” is the native of Tezpur in Assam, India. Numerous varieties of chilli are grown for vegetables, spices, condiments, sauces, and pickles occupying an indispensable position in Indian diet. Apart from the explicit importance of the crop in the diet, chilli is also used in other forms like medicines and beverages and also as an ornamental plant in the gardens. Nutrition wise these are enriched with high Vitamin A and C content; high iron, potassium, and magnesium content with the ability to boost the immune system and lower the cholesterol levels (Grubben and Mohamed El, 2004). India has been a leading producer, consumer and exporter of chilli especially in dried form. Various varieties of the crop are found in India and its quality varies among the states of the country.

## The host crop-*capsicum annum* L.

Known for over 9500 years, chilli is the native of Southern America and was first cultivated in Peru at around 7500 BC (MacNeish, 1964). It has been the first ever domesticated crop of America. During the course of evolution, three important species of *Capsicum* i.e., *C. annuum, C. frutescens* and *C. chinense* evolved from a common ancestor that grew wildly in the North of the Amazon basin (NW-Brazil, Columbia) spreading to the other parts of America. Initially, people cultivated them with other crops in order to protect their crop from the damage caused by birds. By the end of eighteenth century five species of chilli i.e., *C. annuum* L., *C. baccatum* L., *C. chinense* Jacq., *C. frutescens* L., and *C. pubescens* R. & P. were domesticated in different parts of the America (IBPGR, 1983).

Introduction of chilli to India is being credited to the voyage of Columbus, who brought the seeds from Spain, introducing it to Europe, which subsequently spread to Africa and Asia (Heiser, 1976). However, Columbus confused the pungent fruits of *Capsicum* with black pepper *Piper nigrum* L. calling them as red pepper owing to the red colored fruits. *Capsicum* is not related to the *Piper* genus. The popularity of the crop extended swiftly across Europe is moving to India, China and then to Japan. It was incorporated into all the Asian and European cuisines almost instantaneously unlike the other spices introduced from the Western Hemisphere. Since its introduction, it has been cultivated aggressively in all the tropical and sub-tropical countries.

In its course of evolution staring from Central America to the whole of Europe, Africa and Asia, copious terminologies have been used to name the pungent fruits. The phraseology in case of *Capsicum* is however very confusing. Names like pepper, chili, chile, chilli, aji, paprika are used to denote the pungent fruits of *Capsicum*. The term “*Capsicum*” is reserved for taxonomic discussion. The vernacular names include pepper, chile, and aji. The common term used to denote chilli in India is “Mirchi” in Hindi. Bell pepper generally refers to the blocky non pungent fruits of *Capsicum* known as “Shimla Mirch” in Hindi.

**Figure 1:**
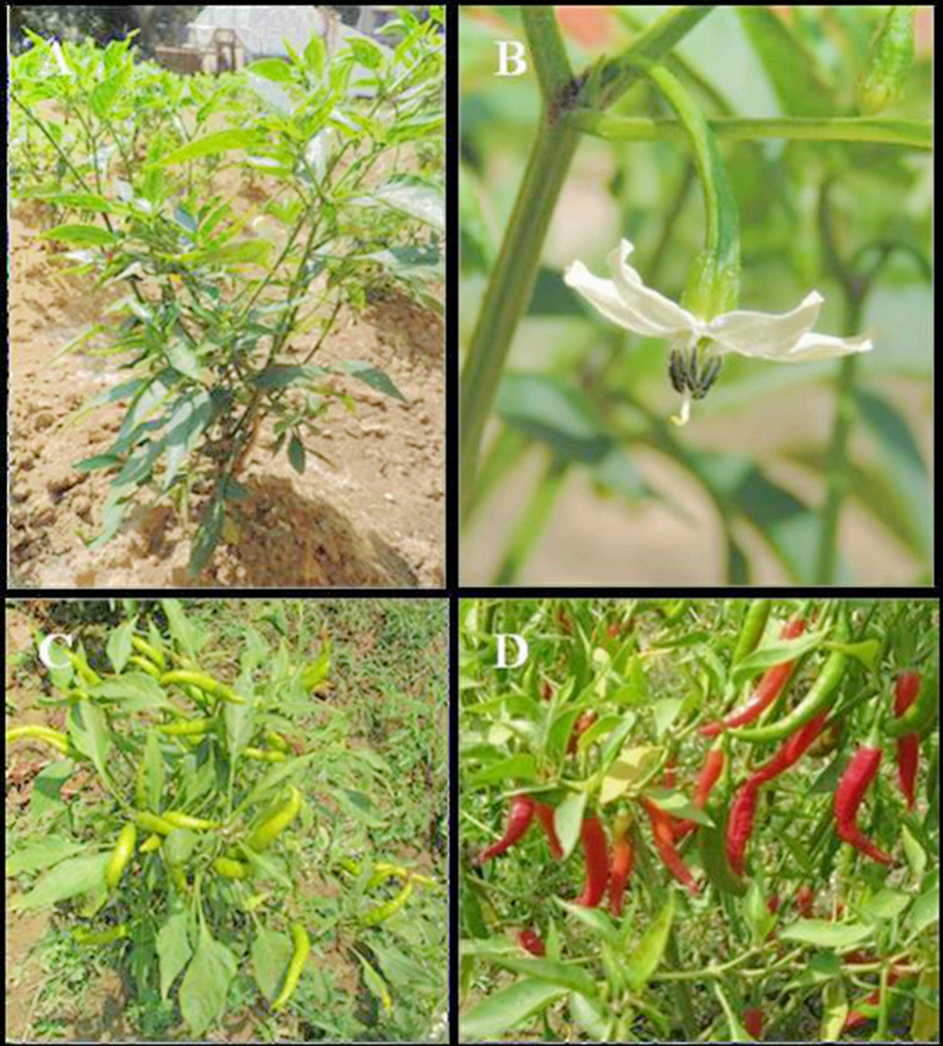
**The botanical characteristics of *Capsicum annum* L., the chilli plant (A), the flower (B), immature green fruits (C) and the ripe red fruits (D)**.

Belonging to the Solanaceae family, *Capsicum* genus consists of approximately 22 wild species and five domesticated species (Bosland, 1994). Having chromosome number 2*n* = 24, *Capsicum* species may be herb or sub-shrub of height up to 2.5 m with extensively branched stem having hairy growth with purplish spots near the nodes. The tap root is strong with numerous lateral roots. Flowers are generally solitary, terminal, bisexual and pentamerous with campanulate to rotate corolla. Stamens are adnate at the base of the corolla tube with blue to purplish anthers. The ovary is superior having 2–4 chambers. Filiform style is found with capitate stigma. Chilli fruits are considered vegetable and are botanically berries (Figure 1).

Based on the fruit characteristics, i.e., color, pungency, shape, flavor, size, and their use, the types of chilli are classified (Bosland, 1992, 1996). It is a perennial crop and can be grown throughout the year. The major harvest season is between December and March with its supply reaching maximum during February-April (Bosland, 1996).

## Uses and importance

Chilli is used in all forms starting from fresh green fruits with ripe fruits along with its dried and powdered form. Fresh green pungent fruits are generally used in salads, stuffing, and as a flavoring agent in cooked meals. The non-pungent varieties are cooked as vegetables or processed with other food items for flavor (Welbaum, 2015). Very pungent varieties are consumed in small quantities generally considered as a condiment or spice for seasoning and for stimulating appetite. Hot peppers are also pickled in salt and vinegar used in ketchups as flavoring agents (Grubben and Mohamed El, 2004). Apart from its extensive use in preparation of processed food, chilli varieties are also used as coloring agents in salad dressings, meat products, cosmetics, and even clothing.

*Capsicum* possesses various medicinal and nutritional values as well. It is interesting to quote that fresh green chilli fruits contain more Vitamin C than found in citrus fruits, while red chilli fruits have more Vitamin A content than as found in carrots (Osuna-García et al., 1998; Than et al., 2008). The active ingredient of the spice, capsaicin is a complex of capsaicinoid alkaloids found in variable concentration in different chilli varieties. It is found in abundance in the placental tissues and cross walls of the fruits. However, in very pungent fruits, it is distributed in all the fleshy parts of the fruit (Grubben and Mohamed El, 2004). The amount of capsaicin has been the measure of the pungency of the chilli variety and is generally expressed in Scoville Heat Units (SHU) (Scoville, 1912).

Capsaicin possesses the antioxidant, anti-mutagenic, anti-carcinogenic, and immunosuppressive activities having the ability to inhibit bacterial growth and platelet aggregation. It is also used as anti-arthritic and anti-inflammatory agent. Regular consumption of chilli fruit is helpful against hemorrhoids, varicose veins, anorexia, and liver congestion. Pure and processed form of chilli extracts is used externally as rubefacient and analgesic in case of back pain, rheumatism, articular and muscular pains and swollen feet and even as antidote in case of poisoning. The two non-foods and non-pharmacological application of capsaicin are in the preparation of “pepper sprays” as weapons of self-defense and in preparation of “natural and organic pesticide” (Welbaum, 2015).

## Global production and indian share

**Figure 2:**
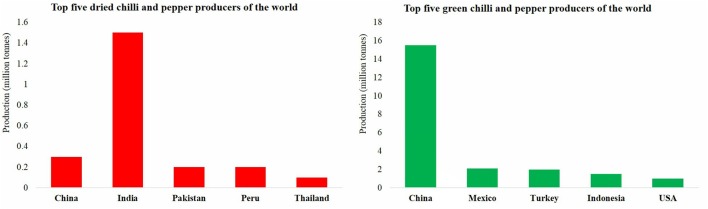
**Production of chilli and pepper (dried and green) in top five chilli producing countries in the world (FAOSTAT, 2011)**.

Being an important crop of tropical and sub-tropical countries, cultivated in large areas of Asia, Africa, South, and Central America and southern Europe, the total area under cultivation in the world is about 1.9 million hectares (Vanitha et al., 2013). About 42.2% (801,600 ha) cultivable land of the total area under chilli production at world level lies in India, producing approximately 21.4% (1.4 million t) of world's total chilli production (FAOSTAT, 2012). India has been the major producer as well as exporter of the dried chilli in the world (Figure 2). The other important producers of chilli include China, Mexico, Peru, Turkey, Thailand, Indonesia followed by countries in tropical Africa mainly Ghana and Ethiopia.

Chilli is a universal spice crop of India grown in almost all the states of the country. The quality of the chilli varies from state to state. For instance, chilli of Karnataka is known for its oil content, Gujarat quality is majorly known for its sharp color while that of Rajasthan is well known for making pickles. The chilli cultivated in Assam is famous for its pungency and that grown in Andhra Pradesh is mostly used as vegetables and liked by non-vegetarians. Major chilli producing states include Andhra Pradesh, Maharashtra, Karnataka, and Madhya Pradesh (Post harvest profile of chilli, 2009). Apart from being a large consumer and producer of chilli, India is also the largest exporter of the crop. Over 30% of the chilli produced in India is exported to countries of West Asia, East Asia, USA, Sri Lanka, and Bangladesh, most commonly in dried form (FAOSTAT, 2012).

## Constraints in chilli production

Though being an important spice crop grown worldwide, many constraints decrease production, causing significant reduction in yield and seed production. Plant diseases have been a major reason for the crop losses worldwide. Diseases caused by fungi, bacteria, viruses or nematodes have adversely affected chilli production in almost all parts of the world. A summary of the various diseases associated with the host crop chilli is outlined in Table 1.

**Table 1:** **Diseases of chilli reported from different parts of the world**.

**S. No**.	**Disease**	**Causal organism**	**Symptoms**	**Plant parts affected**	**Countries affected**	**References**
**FUNGAL DISEASES**						
1.	Fruit Rot	*Colletotrichum truncatum* (*capsici) Colletotrichum gleosporoides Colletotrichum acutatum*	Water soaked and sunken lesions with characteristic rings of acervuli in concentric rings	Leaf, stem, and fruits	Tropical and sub-tropical countries	Than et al., 2008; Saxena et al., 2014
2.	Cercospora leaf spot or velvet spot	*Cercospora capsici Cercospora unonidicola*	Small brown and circular lesions with light gray center and dark margins	Leaf, stem	Worldwide with most severe in warm and moist conditions	Cerkauskas, 2004
3.	Phytophthora blight	*Phytophthora capsici*	Affects root and lower portion of the stem leading to wilting	Leaf, stem, and fruits	South Korea and countries with high humidity and summer rainfall	Sanogo and Carpenter, 2006
4.	Damping off	*Pythium* spp. *Fusarium* spp. *Sclerotinia* spp.	Death of seedlings and subsequent reductions of plant stands	Roots and crown of older plants	Worldwide	Koike et al., 2007
5.	Wilting	*Verticillium dahliae*	stunting, defoliation, and wilting, with discoloration of the vascular system	Whole plant	World wide	Sanogo, 2003
6.	Powdery mildew	*Leveillula taurica*	Chlorotic blotches and spots on leaves followed by their shedding	leaves	Places with warm and dry climate	Glawe et al., 2010
7.	Root rot	*Rhizoctonia solani*	wilting and death	Lower region of stem and root	Worldwide	Sanogo, 2003
8.	Fusarium wilt	*Fusarium solani* var. *capsici*	leaf chlorosis, vascular discoloration, and wilting	leaf	New Mexico	Crawford, 1934
**BACTERIAL DISEASES**						
1.	Bacterial wilt	*Ralstonia solanacearum*	Browning of roots and lower part of stem leading to wilting of plant	Root, Stem	Tropical and subtropical countries with high rainfall	Nguyen and Ranamukhaarachchi, 2010
2.	Bacterial spot	*Xanthomonas campestris* pv. *vesicatoria*	Water soaked lesions on leaves that turn brown, patches on fruits and stem	Leaves, stem, and fruit	Tropical and sub-tropical countries	Abbasi et al., 2002
3.	Bacterial canker	*Corynebacterium michiganense*	Light brown and raised lesions	Leaves, stem	USA	Ivey and Miller, 2000
4.	Bacterial soft rot	*Erwinia carotovora*	Softening of tissues	Fruits	Areas with wet and cold climate conditions	Stommel et al., 1996
**VIRAL DISEASES**						
1.	Pepper leaf curl virus (PLCV)	Whitefly transmitted Geminivirus	Extensive yellowing of leaves with stunted growth	Leaves, stem	India, United States, Nigeria, South Asian Countries	Chattopadhyay et al., 2008
2.	Pepper veinal mottle virus (PVMV)	Aphid transmitted Potyvirus	Veinal and intraveinal chlorosis with stunted leaves and fruits	Leaves, Fruits	Afghanistan, Africa, and India	Berger et al., 2005; Arogundade et al., 2012
3.	Alfalfa mosaic virus (AMV)	Aphid transmitted Bacilliform virus	Distinct white to yellow calico pattern mosaic on leaves	Leaves	New Zealand	Fletcher, 1983
4.	Pepper mottle virus (PeMV)	Aphid transmitted Potyvirus	Mottled leaves with green vein banding and distortion	leaves	Florida, Arizona, Southern USA, Mexico, Central America, India, Thailand	Kaur et al., 2014
5.	Beet curly top virus (BCTV)	Leaf hopper transmitted Geminivirus	Stunted and yellowed plants	Whole plant	Western United States, Eastern Mediterranean basin	Stanley, 2008
6.	Pepper severe mosaic virus (PepSMV)	Aphid transmitted Potyvirus	Leaf shedding, necrotic streaks and spots on fruits, stem, and leaves	Leaf, stem, fruits	Argentina	Ahn et al., 2006
7.	Chilli veinal mottle virus	Aphid transmitted Potyvirus	Mottled leaves with green vein banding, mottled, and distorted pods	Leaf, fruits	Asian countries	Moury et al., 2005
**NEMATODE INFECTIONS**						
1.	Root knot	*Meloidogyne incognita*	Stunted growth, low flowering and yield	Roots, fruits	Different parts of the world	Thiyagarajan and Kuppusamy, 2014
**INSECTS AND MITE INFECTION**						
1.	Mite feeding injury	*Polyphagotarsonemus latus*	“Inverted spoon” shaped leaves, Pods with rusty/corky surface	Leaves, fruits	Australia, Asia, Africa, Europe, North and South America, Pacific Islands	Venzon et al., 2008
2.	Thrips feeding injury	*Thrips parvispinus Scirtothrips dorsalis*	“Boat shaped” curled leaves, distorted pods	Leaves, fruits	India, Sri Lanka, Orient and Pacific Islands, Continental USA	Maharijaya et al., 2011; Johari et al., 2014
3.	Aphid feeding injury	*Myzus persicae Aphis gossypii*	Distorted, mottled young leaves, chlorosis, leaf drop, reduced fruit size	Leaves, fruits	World wide	Tapia et al., 2008; Varghese and Mathew, 2012

Other than the losses due to the pests and pathogens, crop loss in post-harvest conditions further add in delimiting the yield and production of the crop (Prusky, 2011). Specifically, in developing countries, the post-harvest losses are more serious owing to poor transportation and storage facilities (Sharma et al., 2009). Moreover, due to the recent strengthening of the food security norms in the international markets, the trade of food contaminated with fungal toxins, mycotoxins has been declared unhealthy for human consumption (WHO, 2002). Species belonging to *Aspergillus* genus like *A. flavus* (Bankole et al., 2004) and *A. parasiticus* (Garcia-Villanova et al., 2004) have been majorly kept responsible for production of harmful aflatoxins B and G, whose contamination in food commodities has led to serious health problems in human, like cancer and aflatoxicosis (Jeffrey and Williams, 2005). Mycotoxin contamination in dried chillies has limited its export to major export destinations like the UK and USA. Post harvest loss due to aflatoxin contamination in chilli has been reported to be about 20% to about 100% of samples obtained from Turkey (Demircioglu and Filazi, 2010) and Malaysia (Reddy et al., 2011).

Among the large number of diseases affecting chilli cultivation, anthracnose disease caused by *Colletotrichum* species, bacterial wilt by *Psuedomonas solanacearum* and viral diseases like chilli veinal mottle virus (CVMV) infection and cucumber mosaic virus (CMV) infection have been most detrimental to chilli production (Than et al., 2008).

## Anthracnose disease of chilli

**Figure 3:**
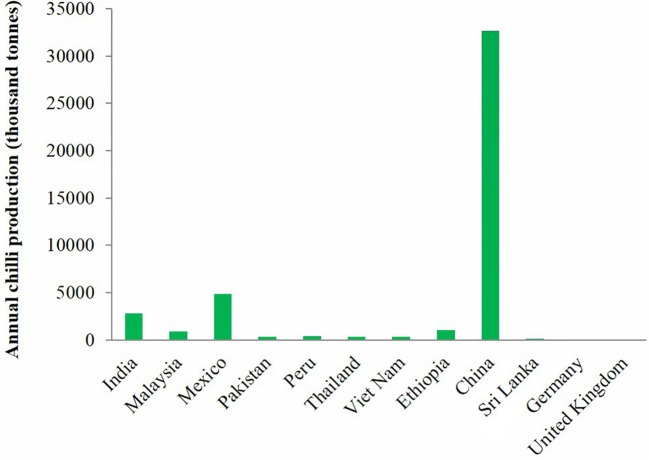
**Annual chilli production by world top producers (FAOSTAT, 2012)**.

Anthracnose disease has been reported to be a major constraint in chilli production in tropical subtropical countries causing huge losses. India had been the largest producer and exporter of chilli, but since a few years the production has declined significantly and presently, India stands at the third number in terms of chilli production (FAOSTAT, 2012; Figure 3). An estimated annual loss of about 29.5%, amounting whopping figure of US$ 491.67 million has been reported from India alone (Garg et al., 2014). In India, a calculated loss of 10–54% has been reported in yield of the crop due to the anthracnose disease (Lakshmesha et al., 2005; Ramachandran and Rathnamma, 2006). Significant losses have been reported from other parts of the world as well, like a significant amount of 20–80% loss has been accounted from Vietnam (Don et al., 2007) and about 10% from Korea (Byung, 2007). The loss is high owing to the post and pre harvest involvement of the pathogen causing a loss of 10–80% of the marketable yield of chilli fruits (Than et al., 2008).

The fruit lesion is the most economically important aspects of the disease as sometimes, even a small lesion on the fruit is enough to lower its market value thereby affecting the profitable yield of the crop (Manandhar et al., 1995). The disease is reported to affect almost all aerial parts of the plant. Chiefly, it causes fruit rot at both green and red stages primarily attacking ripe fruits, hence is also known by the name ripe fruit rot of chilli (Agrios, 2005). The disease is seed borne, soil borne, water borne and airborne and hence may lead to damage at the seedling stage or on the aerial parts of the plants. Many species of *Colletotrichum* have been associated with the pepper anthracnose in different countries. Table 2 gives the brief outline of the different *Colletotrichum* species reported to be associated with the anthracnose of chilli in different parts of the world. However, in India, primarily three important species, namely, *C. capsici, C. acutatum* and *C. gleosporoides* have been reported to be linked with the disease, with *C. capsici* Syd. Butler and Bisby causing major damage at the ripe fruit stage of the plant (Ranathunge et al., 2012; Saxena et al., 2014).

**Table 2:** **Species of *Colletotrichum* associated with anthracnose of chilli in different parts of the world**.

**S.No**.	**Country**	**Species associated**	**References**
1.	Australia	*C. brisbanense*	Damm et al., 2009
2.	Brazil	*C. boninense*	Tozze and Massola, 2009
3.	India	*C. capsici, C. acutatum*	Ranathunge et al., 2012; Saxena et al., 2014
4.	Indonesia	*C. acutatum, C. gloeosporioides, C. nymphaeae, C. capsici*	Damm et al., 2009; Voorrips et al., 2004
5.	Korea	*C. acutatum, C. gloeosporioides, C. coccodes, C. dematium*	Park and Kim, 1992
6.	Mexico	*C. capsici*	Damm et al., 2009
7.	New Zealand	*C. kartsii, C. novae-zelandiae, C. nigrum, C. coccodes*	Damm et al., 2012b; Liu et al., 2013
8.	Papua New Guinea	*C. capsici, C. gloeosporioides*	Pearson et al., 1984
9.	Sri Lanka	*C. acutatum*	Damm et al., 2012a
10.	Taiwan	*C. acutatum, C. capsici, C. gloeosporioides*	Manandhar et al., 1995
11.	Thailand	*C. acutatum, C. capsici, C. gloeosporioides, C. siamense, C. scovillei, C. asianum*	Than et al., 2008; Damm et al., 2009; Phoulivong et al., 2012; Weir et al., 2012
12.	United States	*C. capsici, C. gloeosporioides, C. acutatum, C. coccodes*,	Harp et al., 2008
13.	Vietnam	*C. acutatum, C. capsici, C. gloeosporioides, C. nigrum*	Don et al., 2007
14.	Zimbabwe	*C. nymphaeae*	Damm et al., 2009

## Epidemiology and disease symptoms

Environmental factors play an important role in deciding the severity and spread of any disease. The favorable host, pathogen and weather conditions lead to establishment of disease (Agrios, 2005). Thus, before proposing the management strategy of the disease, a thorough knowledge regarding the epidemiology of the disease should be studied. Anthracnose disease of chilli is generally most common among the tropical and sub-tropical countries. Hot and humid environmental conditions support the spread of the disease.

Other important environmental factors governing the severity of the disease include rainfall intensity and duration, humidity, leaf surface wetness and light. Amongst them leaf surface wetness has been directly linked with the severity of the disease owing to the better establishment of the pathogen in respect of germination, attachment and penetration into host tissues (Than et al., 2008). The relationship between the environmental factors like rainfall intensity and duration and the prevailing temperature and humidity along with the crop geometry and inoculum spread leads to possible development of disease as well (Dodd et al., 1992). Temperature also affects the development of the disease and presence of surface wetness and competitive microbiota further favors the disease development (Royle and Butler, 1986). Temperature around 27°C with relative humidity of 80% have reported to be the most optimum conditions for successful establishment of the disease in a given area (Roberts et al., 2001). The development of the disease also depends on the host cultivar, along with its resistance against the pathogen.

**Figure 4:**
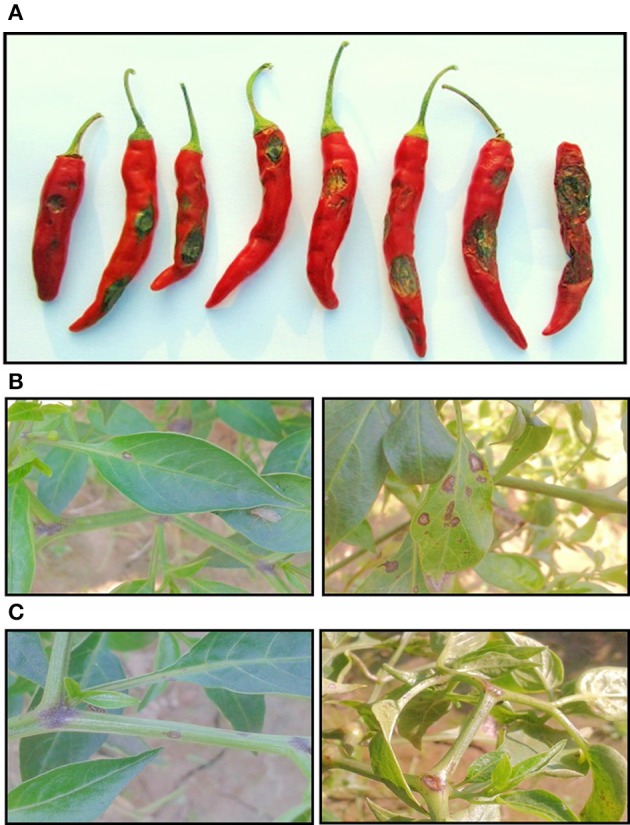
**Characteristic symptoms of anthracnose on chilli fruits (A), leaves (B) and stems (C)**.

On attainment of favorable conditions, characteristic symptoms appear on the ripe chilli fruit, which appear as sunken circular or angular lesions (Figure 4A). Often multiple lesions coalesce to form severe fruit rot. Generally, the lesions are characterized by the presence of black colored spots in concentric rings at maturity. Initially, orange to pink conidial masses may be visible on the fruit surface. The dark spots when observed under a microscope are the acervuli structures containing setae hairs entrapping the conidia of the pathogen. Further, the pathogen forms micro sclerotia in plant debris or seed, soil, which is the mode of survival under unfavorable conditions. The pathogen infects all parts of the host plant, including stems and leaves (Figures 4B,C). Lesions on stems and leaves appear as small sunken grayish brown spots with dark margins, further on which development of acervuli in concentric rings could be easily seen.

## The pathogen-*colletotrichum capsici*

**Figure 5:**
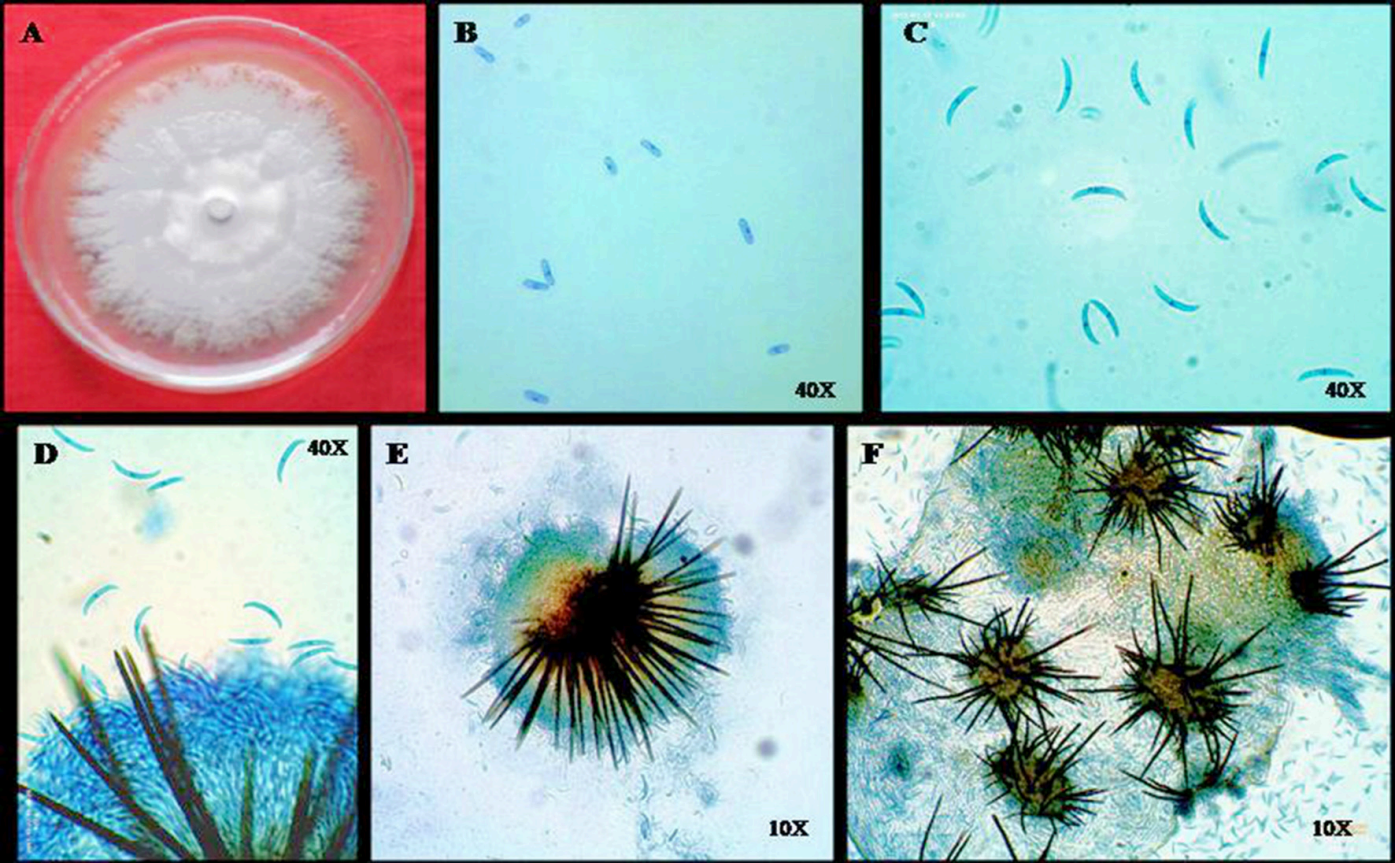
**The morphological appearance of *Colletotrichum* isolates on PDA growth medium (A) and the microscopic appearance of the characteristic structures, i.e., conidia (B,C), setae (D), acervuli (E), and the acervuli as seen on surface of the chilli fruits (F)**.

*Colletotrichum* spp. has been rated as among the ten most notorious pathogens in the world, causing heavy crop losses worldwide (Dean et al., 2012; Figure 5). Specifically, *Colletotrichum* is an asexual genus belonging to phylum Ascomycete and Coeleomycetes class of Fungi imperfectii (Dean et al., 2012). Despite significant developments in studies related to this plant-patho system, the taxonomic position of the pathogen remains unclear. The systematics of the fungal pathogens from this genus still exist in ambiguity with the number of species ranging from 29 to over 700 depending upon the criteria selected for separation (Sutton, 1992).

**Figure 6:**
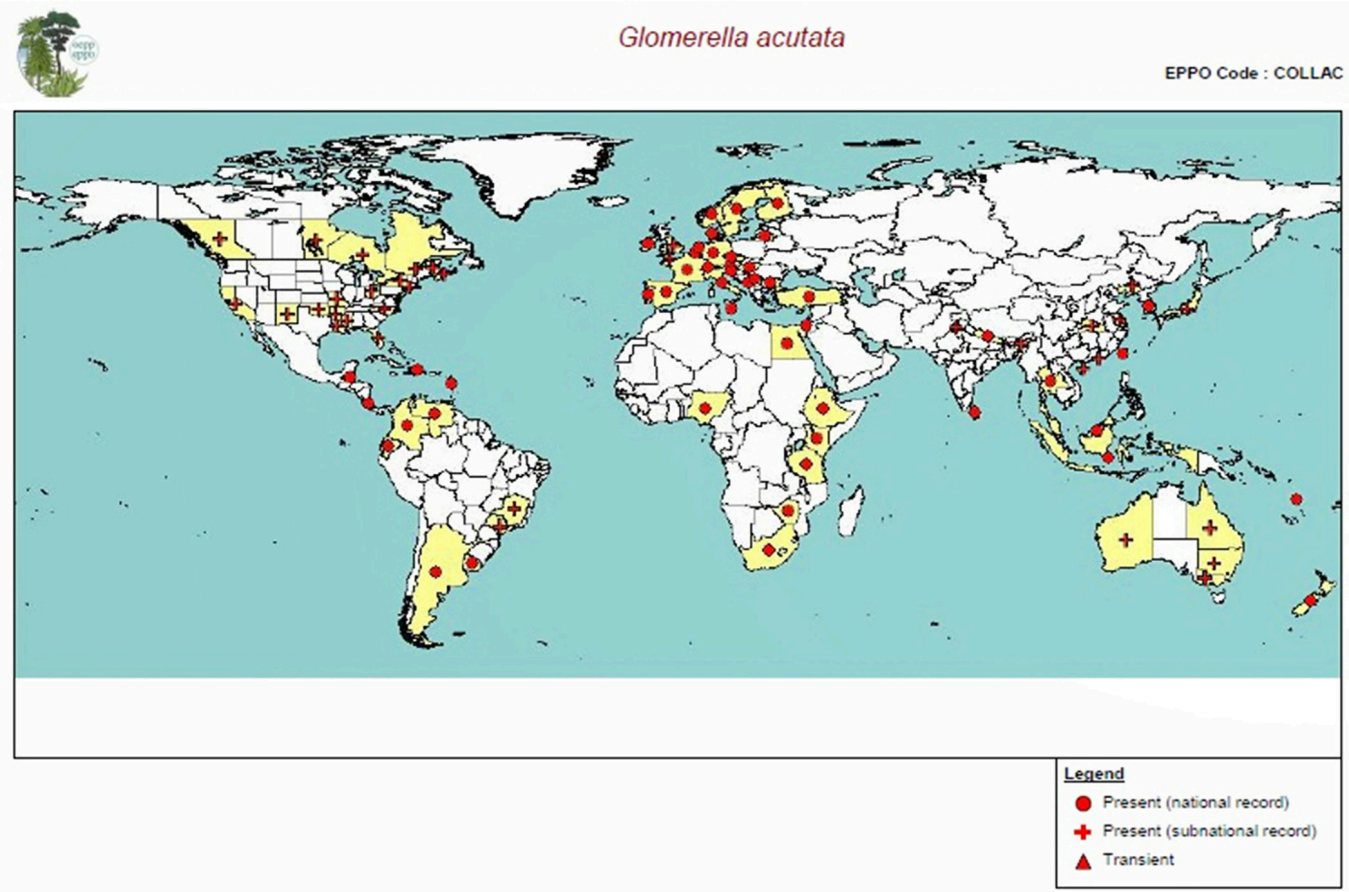
**Distribution map of *Glomerella acutata* (sexual teleomorph of *Colletotrichum acutatum*) (Source: EPPO-PQR)**.

Being economically important pathogen, its host range varies from fruits, vegetables, ornamental plants to important staple food crops. The species of this genus are reported to cause anthracnose disease in more than 121 plant genera from 45 different plant families (Farr et al., 2016). It also causes blights of aerial plant parts and post-harvest rots. The damage may extend to severe economic loss in tropical and sub-tropical countries, causing infection to staple foods like bananas, sorghum, cassavas, legumes, and cereals (Bailey and Jeger, 1992). Particularly, this pathogen exhibit efficient infection in post-harvest conditions owing to its ability to cause latent infection, where the symptoms appear on the fruits only after the harvest or during storage or at the market shelf. Losses up to 100% have been recorded due to *Colletotrichum* spp. (Prusky, 1996). Another important parameter for its successful colonization and severe disease spread may be credited to its cosmopolitan nature. Many species of *Colletotrichum* may be found on a single host or single species may be able to infect different hosts (Sander and Korsten, 2003). Broad, imprecise and often overlapping fungal plant relationships exist in *Colletotrichum* plant-patho system (Freeman and Shabi, 1996). With its ability to infect many hosts along with adapting to new environments, the pathogen poses serious threat to the different crop production system through cross infection problems (Photita et al., 2005). Figure 6 gives the distribution of the sexual teleomorph of the *Colletotrichum* in different parts of the world.

Impact on global economic loss posed by the pathogen has triggered extensive studies on diverse aspects of the biology of the pathogen for better understanding of its infection process and host interaction mechanisms. In light of this, host specificity of the pathogen (Freeman, 2000; Correll et al., 2007) along with the biology involved in the infection mechanisms used by the pathogen (Perfect et al., 1999; O′Connell et al., 2000) and the various fungal-host interactions (Prusky et al., 2000) studies have been carried out. Studies related to genetic diversity and the epidemiology have also been reported (Freeman, 2000; Timmer and Brown, 2000). The genus has been used as a model for studying the genetic basis of symbiotic life styles (Rodriguez, 2000) leading to the development of infection and disease forecasting systems (Uddin et al., 2002). The use of molecular markers like DNA fragments analysis [e.g., Randomly amplified polymorphic DNA (RAPD) and Arbitrarily primed (AP)-PCR] has improved the speed and accuracy in identification and characterization of *Colletotrichum* spp. (Lewis et al., 2004; Photita et al., 2005). Further the the nucleotide sequenceof the 5.8S gene and internal transcribed spacer (ITS) region has facilitated the construction of *Colletotrichum* species specific primers providing a rapid and accurate method for diagnostic purpose and phylogenetic analysis (Torres-Calzada et al., 2011). Many studies have been carried out to resolve the issue of species complex in *Colletotrichum* spp. in different areas of the world on various hosts like mango (Kamle et al., 2013), guava (Mohd Anuar et al., 2014), herbaceous plants (Photita et al., 2005), soursop (Álvarez et al., 2014), and also in medicinal plants as endophytes (Lima et al., 2012) using molecular marker analysis.

The anthracnose causing pathogen in chilli varieties have been reported to be *Colletotrichum capsici* (Sydow), Butler and Bisby (Than et al., 2008). Three pathotypes have been linked with the infection on ripe fruits, while two have been reported causing infection at mature green fruit stages (Mongkolporn et al., 2004; Montri et al., 2009). The convoluted relationships of the fungi with the host have left certain gaps in the knowledge of the infection process and the interactions of different species with chilli plant. Different species have been reported to be linked causing infection in different parts of the chilli plant, for instance, *C*. *acutatum* and *C*. *gloeosporioides* infect the fruits at all stages of development without causing much harm to leaves and stems of the plant, while *C. coccodes* and *C. dematium* mostly cause high infestation on leaves and aerial parts of the plant (Kim et al., 2004). Differential rates of infestation has also been reported by the species of the genus infecting different fruits of the plant. For instance, in Korea, Hong and Hwang (1998) reported *C. gloeosporioides* as the most prevalent species causing an infestation in chilli fruits at both ripe stages and unripe stage; while existence of *C. capsici* and *C. acutatum* as the prevalent species infectionsnfection on ripe and unripe chilli fruits respectively, in the North eastern region of India have been reported (Saxena et al., 2014).

## Infection stages and disease cycle

*Colletotrichum* employs different strategies for causing infection to the host plant which initiate from the intracellular hemibiotrophic mode to the intramural necrotrophic mode of nutrition (Bailey and Jeger, 1992). Liao et al. (2012) has reported an intermediate stage showing partial endophytic life style of the pathogen before adapting to the necrotrophic mode of nutrition in the host plant. Different species of this genus exhibit different mechanism of infection depending on the host infected. For instance, Peres et al. (2005) reported the epiphytic or endophytic mode of survival of *C. acutatum* in an orchard infected with the bitter rot of apple. Also, intramural necrotrophy by *C. capsici* was reported by Pring et al. (1995) while infecting cowpea leading to subsequent necrosis caused due to dissolution of cell wall structures. The biotrophic phase of infection by *C. capsici* is also well studied in the infection caused to broad bean or lentil characterized by the presence of large multilobed, multi septate, vesicular primary hyphae (Latunde-Dada and Lucas, 2007).

Initial infection starts with the attachment of the conidia to the host surface preceded with its germination and production of adhesive appresoria followed with its penetration into the host epidermis. This is further accompanied by the growth and colonization of plant tissue by the fungus, resulting in the formation of specific symptom structures that is acervuli containing the spores of the fungus for further spread (Prusky et al., 2000). The pathogen sometimes remains in quiescent state in the form of appresorial structures in tissues of unripe fruits and cause infection after the fruits ripe or mature (Than et al., 2008). A dendroid structure composed of multiple, thick-walled hyphal branches with swollen or sharp ends from the penetration pore of the appresorium has been recently reported to be an intermediate structure which penetrates the host cuticle layer and infect the epidermal cells during *C. acutatum* infestation in chilli (Liao et al., 2012).

Though the pre-penetration mechanisms exhibited by *Colletotrichum* species appear somewhat related to each other, the post penetration events such as spore adhesion, melanization and cutinization hold certain disparity. Based on the previous studies, four kind of infection strategies with varied hosts have been studied in *C. acutatum* plant patho system (Peres et al., 2005). First is the biotrophic growth of the pathogen, where the formation of appresoria from the conidia is followed by the formation of secondary conidia which further infects and spreads the pathogen inside the host leaves (e.g., The biotrophic disease cycle in citrus leaves). The second is the subcuticular intramural necrotrophy with the development of wide and swollen hyphae in the anticlinal and periclinal walls of host epidermal cells (e.g., The necrotrophic disease cycle on strawberry). The third strategy is the hemibiotrophic mode of infection where the pathogenic hyphae interact with the infection vesicles within the host cells (e.g., The biotrophic disease cycle on blueberry fruits). The fourth type of interaction is the combination of hypertrophic and subcuticular intra and intracellular development of the pathogen generally observed during infestation of almond leaves and fruits.

**Figure 7:**
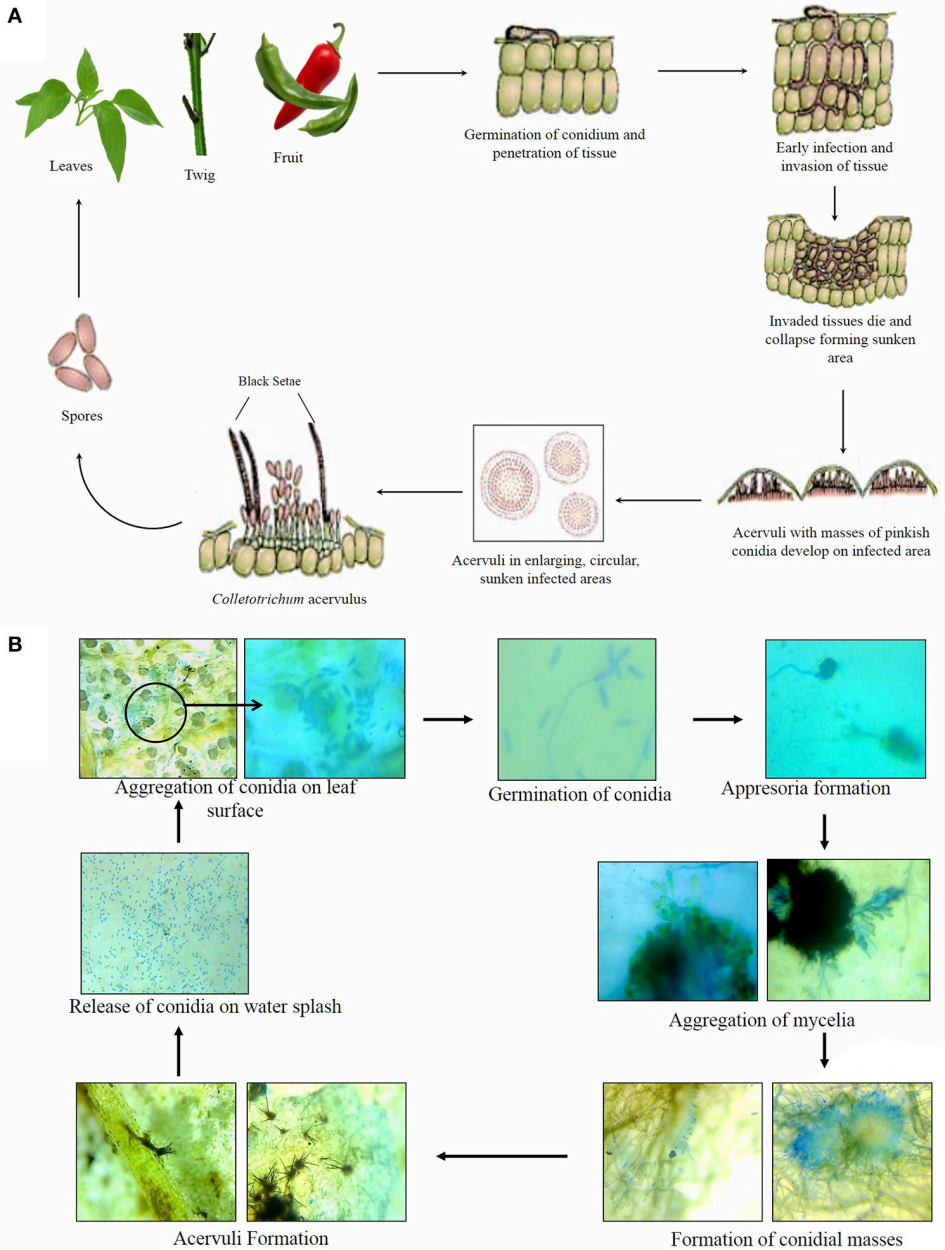
**(A)** Disease cycle of anthracnose disease of chilli (*Capsicum annum* L.) caused by *Colletotrichum* spp. [Source: modified from Agrios (2005)], **(B)** Different stages of infection by the *Colletotrichum* spp. on chilli leaf as seen under microscope.

As far as studies related to infection and colonization by *Colletotrichum* species i.e., *C. gloeosporioides* on susceptible chilli variety is considered, no biotrophic stage in the form of infection vesicle has been found during the infection (Kim et al., 2004). An increased number of small vacuole with the condensed cytoplasm in the epidermal cells followed with cell destruction extending to the sub epidermal cells of the plant due to the action of pathogen enzyme has been noticed during the early stages of infection in chilli plant. During the later stages, inter and intra cellular colonization of the pathogen occurs indicating the governance of necrotrophic mode of fungal growth. Figures 7A,B shows the diagrammatic representation and the microscopic representation of different stages of infection of *Colletotrichum* spp.

## Disease management

Management of chilli anthracnose has been a burning issue for the agriculturists and the farmers as till date, no effective control measures has been proposed. The fall in the chilli production and the drop in fruit quality have further intensified the need for developing a sustainable approach for controlling the spread of the disease. No single management technique has been found to efficiently control the disease. Generally, using a combination of the different strategies like chemical control, biological control, physical control and intrinsic resistance has been recommended for managing the disease (Agrios, 2005). The management strategies for controlling *Colletotrichum* spp. from spreading and establishing a disease can be discussed under four broad categories: Use of cultural Practices, use of chemical control, use of resistant varieties and finally the use of biological control. Table 3, gives a summarized information on the strategies used for controlling the anthracnose disease in chilli from different parts of the world.

**Table 3:** **Control measures for managing chilli anthracnose reported from different parts of the world**.

**Species targeted**	**Active Constituent**	**References**
**CHEMICAL CONTROL**		
*Colletotrichum capsici*	Carbendazim	Than et al., 2008
*Colletotrichum* spp.	Dithiocarbamates, benzimidazole and trizole compounds	Waller, 1992
*C. capsici*	Bavistine (carbendazim)	Ngullie et al., 2010
*C. capsici*	Maneb (Mancozeb)	Smith, 2000
*C. capsici*	Carbendazim, Mancozeb, Trinidazole, Propiconazole	Hegde and Anahosur, 2001
*C. capsici*	Carbendazim, Propiconazole	De los Santos and Romero, 2002
*C. capsici*	Strobilurin fungicides	Lewis and Miller, 2003
*C. gloeosporioides, C. capsici*	Azoxystrobin	Saxena et al., 2016b
*C. capsici*	Thiophanate methyl	Ushakiran et al., 2006
*C. capsici*	Propiconazole and Difenoconazole	Gopinath et al., 2006
*Colletotrichum* spp.	Carbendazim, Mancozeb	Park, 2007
*C. capsici*	Benzimidazole	Kim et al., 2007
*C. capsici*	Azoxystrobin	Chen et al., 2009
*C. gloeosporioides*	Bavistin (carbendazim)	Ngullie et al., 2010
*C. gleosporoides, C. capsici*	Carbendazim	Suwan and Na-Lampang, 2013
*C. capsici*	Difenoconazole	Soytong et al., 2014
**PHYSICAL CONTROL**		
*C. gloeosporioides*	Temperature (25°–30°C)	Denner et al., 1986
*C. capsici*	Rice Straw and Plastic Mulches	Vos et al., 1995
*C. capsici*	Crop rotation	Roberts et al., 2001
*C. capsici, C. acutatum*	Crop rotation, using disease free seeds, fallowing	Agrios, 2005
*C. acutatum, C. capsici*	Good drainage	Than et al., 2008
*C. capsici, C. acutatum, C. gloeosporioides*	Use of resistant plant genotypes	Pollegioni et al., 2012
*C. acutatum*	Light intensity (Green light)	Yu et al., 2013
**BIOLOGICAL CONTROL**		
*C. capsici*	*Trichoderma viride*	Naglot et al., 2015
*C. capsici*	*Psuedomonas fluorescens*	Ramamoorthy and Samiyappan, 2001
*C. gloeosporioides*	Mixture of PGPRs	Jetiyanon and Kloepper, 2002
*C. capsici*	*P. fluorescens, B. subtilis*	Bharathi et al., 2004
*C. capsici*	*P. fluorescens*	Ekbote, 2005
*C. capsici*	*T. harzianum* and *P. fluorescens*	Srinivas et al., 2006
*C. capsici*	*T. harzianum*	Oanh et al., 2006
*C. gloeosporioides*	*T. harzianum*	Jebessa and Ranamukhaarachchi, 2006
*C. capsici*	*Pichia guilliermondi*	Chanchaichaovivat et al., 2007
*C. gloeosporioides*	*T. harzianum*	Boonratkwang et al., 2007
*C. capsici*	*P. fluorescens*	Intanoo and Chamswarng, 2007
*C. acutatum*	*Bacillus subtilis*	Wharton and Diéguez-Uribeondo, 2004
*C. acutatum*	*Candida oleophila*	
*C. capsici*	*P. fluorescens*	Anand et al., 2009
*C. capsici*	*Bacillus subtilis*	Sutarya et al., 2009
*C. capsici*	*Pichia guilliermondii*	Nantawanit et al., 2010
*C. capsici*	*T. viride, P. fluorescens*	Ngullie et al., 2010
*C. gloeosporioides, C. capsici*	Actinomycetes	Intra et al., 2011
*C. acutatum*	*B. vallismortis* strain BS07	Park et al., 2013
*C. capsici*	*Colletotrichum globusum*	Vasanthakumari and Shivanna, 2013
**BOTANICALS AND BIOLOGICAL ELICITORS**		
*C. capsici*	Fungal glucan, Polytran L	Bhandel and Paxton, 1991
*C. capsici*	Neem extract, *Rhinocanthus nasuta* extract, Garlic extract	Singh and Korpraditskul, 1999
*C. capsici*	Extracts of plucao and sabsua	Puttawong and Wongroung, 2009
*C. capsici*	Neem extract, Garlic extract	Ngullie et al., 2010
*C. capsici*	Crude Extract of Piper betle L.	Johnny et al., 2011
*C. capsici*	Extract of *Coleus aromaticus*	Ajith et al., 2012
**INTEGRATED MANAGEMENT**		
*C. capsici*	*T. harzianum*+Captan+Neem Cake	Sharma et al., 2004
*C. capsici*	*P. fluroescens*+Azoxystrobin	Anand et al., 2010

### Use of cultural practices

The pathogen being seed borne, wind borne and water borne apart from being soil borne, the practices to control its spread should target three main areas of disease free crop production in the field: proper drainage, crop rotation and removal of any infected plant parts of the field. Water splashes may easily spread the conidia of the pathogen from infected to uninfected plant parts. Also, relative humidity aids successful colonization of the pathogen. So, the field should have proper drainage and irrigation to prevent the outbreak of the disease. Also, proper distance between the plants should be maintained so as to reduce dense canopy, which gives way for creating moisture (Than et al., 2008). Another important practice is the use of transplants raised from disease free seeds of the chilli variety. The transplants should be kept weed free and away from other solanaceous crops. Ideally, the crop should be rotated after every 2–3 years with crops those are not the host of *Colletotrichum* (Roberts et al., 2001). The use of rice straw and plastic mulches has also been reported for effective control of the disease (Vos et al., 1995).

### Use of chemical fungicides

In the absence of any accurate method of controlling the disease, chemical control has been sought as the most effective measure to control the spread of the disease. The longer time required for developing the resistant cultivar and the short span result of the use of fungicides further popularize this method of controlling the disease specifically for anthracnose disease (Wharton and Diéguez-Uribeondo, 2004). However, the remnant toxic residues of the chemicals in the fruits create hindrance to the expected export of the chilli products to other countries, in turn affecting the economy of the country. Also, relying on a single chemical component result in the development of resistance in the pathogenic isolates, which further augments the difficulty in the management of the disease (Staub, 1991; Than et al., 2008). Traditionally, recommended fungicide for the control of the disease is manganese ethylenebisdithiocarbamate (Maneb) (Smith, 2000) and carbendazim, though the use of both fungicides has been found ineffective under severe disease outbreak. The chemical fungicides generally recommended for controlling anthracnose disease are based on copper compounds, dithiocarbamates, benzimidazole and triazole compounds (Waller, 1992). Newer chemicals like strobilurins based fungicides (e.g., azoxystrobin, pyraclostrobin) have also been used for its management. However, only a few reports are available using this class of fungicide controlling chilli anthracnose under large field trials (Schilder et al., 2001; Lewis and Miller, 2003; Chen et al., 2009).

The effective control through the use of chemical fungicides is possible by the timely application during the critical period favorable for the onset of the disease. Generally, fungicides should be applied at young expanding tissues, including fruits, leaves and flowers to restrict the entry of the pathogen to the plant system (Wharton and Diéguez-Uribeondo, 2004). However, numerous reports on the destructive effects of the use of fungicides on farmers' health, economic status, and toxic contamination of the environment, particularly in developing countries cannot be ignored (Voorrips et al., 2004; Garg et al., 2014). Different classes of fungicides have specific mode of action along with their duration of effect on disease control. So, wise choice of fungicides by the farmers in a particular area, according to prevailing environmental conditions, should be taken into consideration. Rotation of two or more different classes of fungicides is highly recommended for increasing the chance of better protection against the disease in the fields (Förster et al., 2007).

### Use of resistant varieties

Developing resistance against the pathogen in the host is seeking to be the most important and sustainable approach for managing the disease. This strategy not only eliminates the losses caused due to the disease, but also remove the chemical and mechanical expense of the disease control (Agrios, 2005). The principle behind the use of resistant cultivars is to trigger the host defense response that in turn would inhibit or retard the growth of the pathogen involving the use of a single gene pair: a host resistance gene and the pathogen avirulence gene (Flor, 1971). In lieu of the existing biotechnological approach to manage diseases, certain successful resistant varieties of chilli against *C. capsici* have been reported from different parts of the world (Yoon, 2003; Voorrips et al., 2004; Garg et al., 2014). Though, not much success has been sought in developing resistant chilli varieties in the species *Capsicum annum* L., which is the only species grown worldwide (Park, 2007). The two major requirements before proceeding for developing the cultivar is the knowledge of the resistant varieties of *Capsicum* occurring wildly in the region and the different pathotypes of the pathogen found in that region. Many varieties resistant to *Colletotrichum* spp. and information regarding the pathotypes of the pathogen has been reported and is available AVRDC, 2003; Babu et al., 2011). However, the challenging task of resistant breeding is exceptionally difficult in *Colletotrichum*-chilli pathosystem due to the association of more than one species of the pathogen with the disease (Sharma et al., 2005; Saxena et al., 2014) along with the differential ability of the pathogenic virulence (Montri et al., 2009).

Recently, a study carried by Garg et al. (2014) reported the existence of nine resistant varieties (BS-35, BS-20, BS-28, Punjab Lal, Bhut Jolokia, Taiwan-2, IC-383072, Pant C-1, and Lankamura Collection) of *Capsicum* spp. out of the 42 varieties existing in use in the area which could be employed for developing successful resistant cultivars through breeding programs. The information on the resistance varieties against *Colletotrichum* may also be utilized for studying the inheritance of the resistance from one generation to another (Kim et al., 2008) and also to locate and study the quantitative trait loci (QTLs) maps for resistance (Lee et al., 2010).

### Use of botanicals and biological control agents

Disease control through the use of botanicals and crude extracts of medicinal plants have been explored in recent years for their effective antifungal and antimicrobial properties. Their easy decomposition, non-residual activity and non phytotoxic properties further popularize their use for controlling phytopathogens. Several studies using crude plant extracts have also been conducted to access the control of *Colletotrichum* spp. on chilli (Ngullie et al., 2010; Johnny et al., 2011). They have shown different degree of effectiveness of extracts of sweetflag (*Acorus calamus* L.), palmrosa (*Cymbopogon martini*) oil, *Ocimum sanctum* leaf extract, neem (*Azadirachta indica*) oil, garlic, *Piper betle* L., *Coleus aromaticus*, plucao, and sabsua against the pathogen growth and spore germination.

Biocontrol strategy for disease management has stood up as a sustainable approach required for restoring the lost homeostasis of the environment. Though, for managing chilli anthracnose this particular strategy has not gained much momentum yet, the potential of using biocontrol agents (BCAs) for controlling the pathogen was elucidated way back by Lenné and Parbery (1976). The possibilities of using BCAs for controlling the post harvest loss of fruits has been illustrated by Jeger and Jeffries (1988) and Korsten and Jeffries (2000). Till date, the BCAs used for studying the antagonistic potential against *Colletotrichum* spp. affecting chilli crop include *Psuedomonas fluorescens, Trichoderma* spp., *Bacillus subtilis, Candida oleophila*, and *Pichia guilliermondi* (Table 3).

### *Trichoderma* as a biocontrol agent against *colletotrichum* spp.

Belonging to class Ascomycete, *Trichoderma* is a well-studied ubiquitous genus. Well known as a saprophytic fungus, it has high adaptive potential as evident from its ability to colonize wood, bark, agricultural wastes and other substrates apart from its omnipresence in a variety of soil types (Singh et al., 2012; Mukherjee et al., 2014). Its biocontrol potential has been well established against numerous important phytopathogens like *Alternaria, Colletotrichum, Phytophthora, Pythium, Rhizoctonia, Sclerotinia, Verticillium* etc. (Begum et al., 2008; Imtiaj and Lee, 2008; Jain et al., 2012; Singh et al., 2013). The mechanisms involved have been attributed to be mycoparasitim, antibiosis, competition for nutrients and space along with its ability to induce systemic resistance in the plants against the pathogens (Harman, 2006; Shoresh et al., 2010; Hermosa et al., 2012). Also, the efficient enhancement in plant growth with significant increase in biomass has also been attributed to the application of *Trichoderma* species. (Yedidia et al., 2001; Jain et al., 2012). Recently, the ability of the fungus to induce biotic tolerance in plants by enhancing the mechanical strength of the plant system has been studied against phtytopathogenic infestation (Singh et al., 2013; Saxena et al., 2015).

Specifically, in the *Colletotrichum* plant pathosystem, its potential has been elucidated owing to its fast colonizing ability and mycoparasitic nature, which results in coiling and parallel growth of the pathogen (Begum et al., 2008; Živkovic et al., 2010). This property has been further attributed due to the secretion of extracellular enzymes, including glucanases, chitinases etc. that degrade the pathogenic mycelia thereby restricting its growth and further colonization in the host tissue (Harman, 2006; Vinale et al., 2008; Singh et al., 2012). The various studies reporting the application of *Trichoderma* species for the biological control of *Colletotrichum* in different host have been summarized in Table 4.

**Table 4:** ***Trichoderma* mediated control of *Colletotrichum* species**.

**S.No**.	***Trichoderma* species used**	**Targeted *Colletotrichum* species**	**Host plant**	**References**
1.	*T. viride*	*C. dematium*	Pigeon Pea	Kumar et al., 2000
2.	*T. viride*	*C. lindemuthium*	Cow Pea	Adebanjo and Bankole, 2004
3.	*T. harzianum*	*C. acutatum*	Strawberry	Freeman et al., 2004
4.	*T. harzianum*	*C. graminicola*	Maize	Harman et al., 2004
5.	*T. harzianum*	*C. gloeosporioides*	Grape	Soytong et al., 2005
6.	*T. viride*	*C. capsici*	Chilli	Kaur et al., 2006
7.	*T. harzianum*	*C. acutatum*	Blueberry	Verma et al., 2006
8.	*T. harzianum*	*C. dematium*	Soybean	Shovan et al., 2008
9.	*T. harzianum*	*C. acutatum, C. gloeosporioides*	Fruits	Živkovic et al., 2010
10.	*T. harzianum, T. psuedokoningii*	*C. destructivum*	Cow pea	Akinbode and Ikotun, 2011
11.	*T. viride, T. harzianum, T. hamatum*	*C. lindemuthium*	Bean	Padder and Sharma, 2011
12.	*T. viride*	*C. gloeosporioides*	Sarpagandha	Ghosh and Chakraborty, 2012
13.	*T. harzianum*	*C. capsici*	Chilli	Rahman et al., 2013; Saxena et al., 2016a
14.	*Trichoderma* spp.	*C. gloeosporioides*	Mango	Admasu et al., 2014
15.	*T. viride and T. harzianum*	*C. lindemuthium*	Haricot Bean	Amin et al., 2014

The mode of use of this fungus has been restricted to seed biopriming or root treatment of the plants. The effect of the foliar sprays of *Trichoderma* species to prevent spread of foliar disease has not been studied extensively. However, effective results have been reported by the foliar sprays of other antagonistic microbes like yeast, *Psuedomonas, Bacillus* etc. that managed to control the growth and colonization of the pathogenic fungus (Chanchaichaovivat et al., 2007; Anand et al., 2009; Sutarya et al., 2009). The reports have very well elucidated the enhanced efficiency of the microbes to combat the growth of pathogen at leaf and fruit surface acting as a first line of defense for the protection of the host plants. Similar approach for efficient control of foliar disease of *Capsicum* i.e., anthracnose could be proposed by using *Trichoderma* strains as well. There have been reports showing effective potential of *Trichoderma* species in controlling *Colletotrichum* infestation in other hosts like cowpea (Adebanjo and Bankole, 2004). Also report on effective colonization of *Trichoderma* species in the phylloplane of plants is well studied (Bae et al., 2011). In order to control chilli anthracnose very few attempts have been made to use *Trichoderma* isolates obtained from the phylloplane of healthy host plant. The need for an effective all around protection of the plant triggered our group to study the effectiveness of *Trichoderma* isolates dwelling at the phylloplane of the healthy plants to concur the pathogenic ingression. Recently, *Trichoderma* isolates from the phylloplane of healthy leaves were found equally successful in controlling disease incidence as evident from the elevated induction of defense related enzymes and reduced disease incidence on host plants (Saxena et al., 2016a).

## Future prospects

Though the epidemic nature of the disease has been studied for ages, many areas are still unexplored in terms of host-pathogen interaction, its spread and effective management strategies. There lies an urgent need to develop an efficient integrated management strategy keeping in concern the different environmental factors and pathogenic resistance, driving the successful colonization of the pathogen in the host tissues. An insight into the pathogen's lifestyle would provide valuable information required to develop targets for developing resistant varieties of chilli against the pathogen. Also, modifications in conventionally recommended cultural practices suiting to a particular agro-climatic region will prove helpful in better management of the disease. More studies are required for acquiring in-depth information regarding various modes of infection by the pathogen and the pathogenic variability associated within a region with the post-harvest as well as pre-harvest loss in the crop production. The overall knowledge about the key aspects of a disease triangle will enable better management of the disease keeping track of the quality and quantity of the crop produced thereby contributing efficiently to the country's economy.

## Author contributions

AS has drafted the Manuscript; RR, HS, and VG have critically reviewed the draft for important intellectual content and provided substantial contribution for the concept and design of the Manuscript.

### Conflict of interest statement

The authors declare that the research was conducted in the absence of any commercial or financial relationships that could be construed as a potential conflict of interest.
